# Secular trends in stillbirth by maternal socioeconomic status in Spain 2007–15: a population-based study of 4 million births

**DOI:** 10.1093/eurpub/ckz086

**Published:** 2019-05-23

**Authors:** Miguel Angel Luque-Fernandez, Aurielle Thomas, Bizu Gelaye, Judith Racape, Maria Jose Sanchez, Michelle A Williams

**Affiliations:** 1 Andalusian School of Public Health, Biomedical Research Institute of Granada, University of Granada, Granada, Spain; 2 Department of Epidemiology and Population Health, London School of Hygiene and Tropical Medicine, London, UK; 3 Department of Epidemiology, Harvard T.H. Chan School of Public Health, Boston, MA, USA; 4 Public Health School, Centre de Recherche Épidémiologie, Biostatistique et Recherche Clinique, Université Libre de Bruxelles, Brussels, Belgium; 5 Department of Population Medicine, Harvard Medical School, Harvard Pilgrim Health Care Institute, Boston, MA, USA; 6 Biomedical Research Networking Centres of Epidemiology and Public Health (CIBERESP), Madrid, Spain

## Abstract

**Introduction:**

Stillbirth, one of the urgent concerns of preventable perinatal deaths, has wide-reaching consequences for society. We studied secular stillbirth trends by maternal socioeconomic status (SES) in Spain.

**Methods:**

We developed a population-based observational study, including 4 083 919 births during 2007–15. We estimate stillbirth rates and secular trends by maternal SES. We also evaluated the joint effect of maternal educational attainment and the Human Development Index (HDI) of women’s country of origin on the risk of stillbirth. The data and statistical analysis can be accessed for reproducibility in a GitHub repository: https://github.com/migariane/Stillbirth

**Results:**

We found a consistent pattern of socioeconomic inequalities in the risk of delivering a stillborn, mainly characterized by a persistently higher risk, over time, among women with lower SES. Overall, women from countries with low HDIs and low educational attainments had approximately a four times higher risk of stillbirth (RR: 4.44; 95%CI: 3.71–5.32). Furthermore, we found a paradoxical reduction of the stillbirth gap over time between the highest and the lowest SESs, which is mostly due to the significant and increasing trend of stillbirth risk among highly educated women of advanced maternal age.

**Conclusion:**

Our findings highlight no improvement in stillbirth rates among women of lower SES and an increasing trend among highly educated women of advanced maternal age over recent years. Public health policies developing preventive programmes to reduce stillbirth rates among women with lower SES are needed as well as the necessity of further study to understand the growing trend of age-related stillbirths among highly educated women in Spain.

## Introduction

Stillbirths are an important health outcome and a tragedy that deserves attention.^1^ Often, stillbirth rates are used as an indicator of the level of development of a country.^2^ Despite the fact that western countries’ stillbirth rates are lower than those in developing countries, stillbirth rates have remained relatively stable over the last few decades.^3^

Higher stillbirth rates have been found in Western countries among women of lower socioeconomic status (SES) and specific ethnic groups.^3–6^ Maternal educational attainment and country of origin at birth have been used by several authors as a proxy for maternal SES.^3^^,^^4^^,^^7^^,^^8^ The use of maternal country of origin at birth, in this way, is supported by studies which have shown evidence of worse perinatal outcomes among immigrant women in high-income countries.^9^^,^^10^

Europeristat, a European report focussing on perinatal health, recommended that stillbirth rates should be used as an indicator to allow international comparisons.^11^ Stillbirth risk provides information on avoidable mortality and reveals problems in the quality of perinatal care. Secular trends in perinatal mortality have been used as a public health indicator, as it is highly sensitive to social and health inequalities.^12^

We described stillbirth secular trends by maternal country of origin at birth, the 2015 Human Development Index (HDI) of their respective country of origin and maternal educational attainment as proxies of maternal SES during the period 2007–15 in Spain. Secondarily, we explored joint effects on stillbirth of (i) low maternal educational attainment and advanced maternal age and (ii) low maternal educational attainment and low HDI.

## Methods

### Type of study and data source

We designed a population-based observational study using vital statistics from Spain’s National Institute of Statistics (INE). The final dataset contains basic sociodemographic, obstetric and perinatal information for 11 323 stillbirths and 4 179 402 total births in Spain from 2007–15. Infants born before 28 gestational weeks were excluded (Supplementary figure S1).

### Main outcome and exposures

The main outcome of the study is stillbirth, which is defined as foetal death at ≥ 28 gestation weeks at delivery.^13^ The main exposure of interest was maternal SES. We used maternal educational attainment at the country of birth, maternal country of origin at birth and the 2015 HDI from the women’s country of origin as proxies of maternal SES. The maternal educational attainment refers to the highest degree obtained by the woman at the time of delivery and is based on the International Standard Classification of Education.^14^ Maternal educational attainment was categorized as (i) secondary education or lower (mothers with ≤ 12 years of the mandatory education in Spain), (ii) upper secondary and first stage of tertiary education (mothers with >12 but ≤ 15 years of education) and iii) tertiary education (mothers who received >15 years of education). The HDI quantifies the social progress of countries, based on a summary measure of average achievement in key dimensions of human development: a long and healthy life, being knowledgeable, and a decent standard of living. The index is quantified as the geometric mean of normalized indices for each of the three previous dimensions.^15^ We categorized the HDI into four groups: low, medium, high and very high HDI.^15^ Finally, we categorized maternal country of origin into six groups: Africa, America and the Caribbean, Asia and Oceania, other European Union countries with 15-member states (EU15), non-EU15 European Countries, and Spain.^11^

### Other variables

Parity, calendar year, maternal age (years) at the time of delivery were included in the statistical analysis. Parity was dichotomized as nulliparous (women who have never previously given birth) and multiparous (women who have ≥ 1 prior births). Maternal age was categorized consistently based on international recommendations as follows: ≤19, 20–24, 25–29, 30–34, 35–39 and ≥40 years.^16^

### Statistical analysis

In descriptive analyses, we summarized the total number of births and stillbirths by baseline characteristics and calendar year. Next, we computed stillbirth rates and univariate rate ratios with 95%CIs by population characteristics. We also computed linear trend tests for stillbirth rates by maternal country of origin and educational attainment over the time-period in analysis. Furthermore, we computed stillbirth rates of change (percent) for maternal educational attainment by calendar period and categories of maternal age (<35, 35–39 and ≥40 years).

In multivariable analyses, we used generalized linear regression models with family Poisson and link log to derive rate ratios. We derived ‘sandwich’ standard errors for statistical inference.^17^ The final multivariable model was adjusted for maternal age, educational attainment, parity, HDI and calendar period. We used this model to estimate the joint effect on stillbirth of maternal educational attainment and maternal age at delivery. Age was introduced in the model with four categories (<25, 25–29, 30–34 and ≥35) as we combined the categories of age ≥19 and 20–24 given the reduced number of women with tertiary studies among this group of age. Similarly, we also assessed the joint effect on stillbirth of maternal educational attainment and HDI (very high, high, medium and low). Based on the linear combination of the coefficients we derived rate ratios for each of the categories of the linear combination considering women with tertiary education, very high HDI and 25–29 years the reference group against which, women in the other groups were compared.

Data were analyzed using the statistical software Stata Multi-Processor Parallel Edition v.15 (StataCorp, College Station, TX, USA) and R v.3.3.4 (R Foundation for Statistical Computing, Vienna, Austria). The data and statistical analysis can be accessed for reproducibility in a GitHub repository: https://github.com/migariane/Stillbirth

## Results

During the 9-year study period (2007–15), 4 207 372 infants were born in Spain. We excluded 11 450 births <28 gestation weeks and 16 520 (0.4%) births not linked to the 2015 HDI. The final dataset included 4 179 402 births (11 323 stillbirths and 4 168 09 live births) (Supplementary figure S1).

Overall, stillbirth rates per 1000 births were higher among very young (≤19 years) and those of advanced reproductive age (≥35 years). Furthermore, stillbirth rates were higher among women with an African country of origin, with low educational attainment, and low HDI, respectively. Stillbirth rates were approximately two times higher risk for African women, low educational attainment, and low HDI. Women from low HDI countries showed approximately three-times higher risk of stillbirth with a univariate rate ratio of 2.8 (95%CI 2.4–3.3). There was strong evidence of an increasing linear trend in stillbirth risk across levels of the variables maternal age, educational attainment and HDI (table 1, Supplementary table S1). However, no evidence of a secular trend by calendar period over the 9-year period was observed (test of linear trend *P* values = 0.946) (table 1).

**Table 1:** Univariate stillbirth rate among women at least 28 weeks’ gestation by maternal age, country of origin, SES, parity and period in Spain, during 2007–15 (11 323 stillbirths and 4 179 402 total births)

Variables	Total births (*n*)	Stillbirths (*n*)	Rate per 1000 births (95%CI)	Rate ratio (95%CI)	*P*-value
Maternal age in years					<0.001
≤19	100 082	321	3.2 (2.9, 3.6)	1.17 (1.02, 1.35)	
20–24	355 059	1015	2.8 (2.7, 3.0)	1.0 (Reference)	
25–29	845 889	2218	2.6 (2.5, 2.7)	0.93 (0.85, 1.00)	
30–34	1 561 448	3781	2.4 (2.3, 2.5)	0.85 (0.79, 0.92)	
35–39	1 070 715	3093	2.9 (2.7, 3.0)	1.03 (0.95, 1.11)	
≥40	234 886	895	3.8 (3.6, 4.1)	1.37 (1.24, 1.52)	
Parity					<0.001
Nulliparous (first delivery)	2 106 505	6373	3.0 (2.9, 3.1)	1.18 (1.13, 1.23)	
Multiparous (≥1 deliveries)	2 061 574	4950	2.4 (2.3, 2.5)	1.00 (Reference)	
Maternal country of origin					<0.001
Spain	3 372 357	8545	2.5 (2.4, 2.6)	1.00 (Reference)	
EU15	60 990	142	2.3 (2.0, 2.7)	0.93 (0.77, 1.11)	
Other European countries	160 896	435	2.7 (2.5, 3.0)	1.10 (0.99, 1.23)	
Africa	264 356	1290	4.9 (4.6, 5.1)	2.31 (2.16, 2.47)	
America	248 056	720	2.9 (2.7, 3.1)	1.20 (1.11, 1.31)	
Asia and Oceania	61 424	191	3.1 (2.7, 3.6)	1.84 (1.58, 2.14)	
HDI^a^ for maternal country of origin (2015)					<0.001
Low	33 686	176	5.2 (4.4, 6.0)	2.78 (2.36, 3.27)	
Medium	340 616	1430	4.2 (4.0, 4.4)	1.96 (1.84, 2.09)	
High	204 019	602	2.9 (2.7, 3.2)	1.24 (1.14, 1.36)	
Very high	3 589 758	9115	2.5 (2.4, 2.6)	1.00 (Reference)	
Maternal education attainment^b^					<0.001
Secondary education or lower	2 396 836	7087	3.0 (2.9, 3.1)	2.36 (2.19, 2.54)	
Upper secondary or first stage of tertiary	965 730	1413	1.5 (1.4, 1.5)	1.12 (1.02, 1.23)	
Tertiary education	719 034	906	1.3 (1.2, 1.3)	1.00 (Reference)	
Period					0.946
2007	491 288	1099	2.7 (2.5, 2.8)	1.00 (Reference)	
2008	516 602	1409	2.7 (2.6, 2.9)	0.98 (0.91, 1.06)	
2009	491 856	1344	2.7 (2.6, 2.9)	0.96 (0.91, 1.05)	
2010	483 456	1303	2.7 (2.5, 2.8)	0.95 (0.87, 1.03)	
2011	468 936	1288	2.7 (2.6, 2.9)	0.99 (0.91, 1.07)	
2012	451 678	1259	2.8 (2.6, 2.9)	1.01 (0.92, 1.09)	
2013	422 742	1188	2.8 (2.6, 3.0)	1.00 (0.92, 1.09)	
2014	424 498	1111	2.6 (2.5, 2.8)	0.95 (0.87, 1.04)	
2015	417 023	1112	2.7 (2.5, 2.8)	0.98 (0.89, 1.06)	

a HDI (UNESCO).

b 2.1% (88 396) missing values for maternal education attainment.

**Figure 1:**
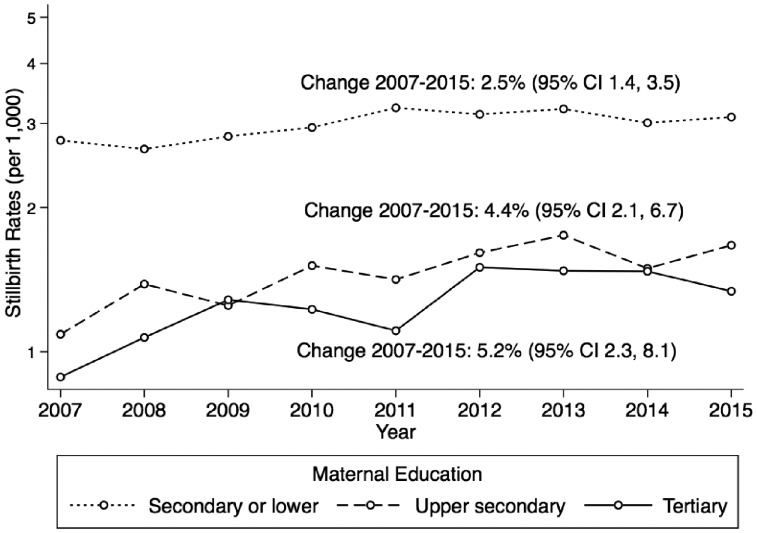
Stillbirth rate of births ≥28 gestational weeks by maternal education in Spain from 2007 to 2015 (11 113 stillbirths and 4 083 919 total births)

Regarding women’s country of origin, there was no evidence of a secular trend in stillbirth risk for any of the other five maternal regions of origin including native women (test of linear trend *P* values = 0.342; Supplementary figure S2). However, there was strong evidence of a secular trend by maternal educational attainment (test of linear trend *P* values < 0.001) (figure 1).

**Figure 2:**
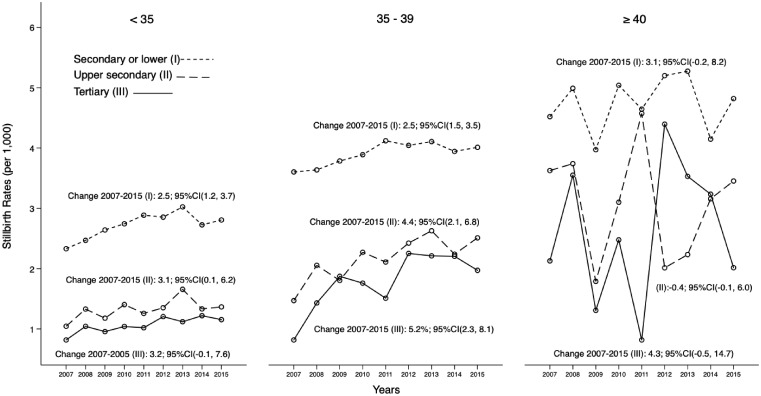
Stillbirth rate of births ≥28 gestational weeks by maternal education and age in Spain from 2007 to 2015 (11 113 stillbirths and 4 083 919 total births)

The gap in stillbirth rates between the highest and lowest levels of maternal education attainment decreased over the 9-year period; mostly because the average rate of change of stillbirth rates was approximately two times higher among women with tertiary education than among mothers with secondary or lower educational attainment: 5.2% (95%CI: 2.3–8.1%) vs. 2.5% (95%CI: 1.4–3.5%) (figure 1). Among highly educated women (tertiary education), the rate of change of stillbirth was 1.6 times higher for women aged 35–39 years compared with the rate of change of women <35 years of age (rates of change: 5.2%; 95%CI: 2.3–8.1 and 3.2%; 95%CI: −0.1–7.6%, respectively). Furthermore, the rate of change was also greater for women aged ≥40 compared with the rate of women aged <35 years, 4.3 vs. 3.2%, respectively (figure 2).

In the multivariable analysis, low HDI, low educational attainment and advanced maternal age were strongly associated with higher risk of stillbirth, showing approximately two times higher risk than women from countries with very high HDI, tertiary education and aged between 20 and 24 years (table 2, model 5).

**Table 2:** Stillbirth rate ratios comparing the HDI for maternal countries of origin adjusted for maternal age, SES, parity and period in Spain, during 2007–15 (11 323 stillbirths and 4 179 402 total births)

Variables	Model 1	Model 2	Model 3	Model 4	Model 5
HDI					
Low	2.05 (1.76, 2.38)	2.10 (1.80, 2.43)	1.67 (1.40, 1.96)	1.74 (1.47, 2.05)	1.73 (1.46, 2.04)
Medium	1.65 (1.56, 1.75)	1.69 (1.60, 1.78)	1.33 (1.24, 1.42)	1.38 (1.29, 1.46)	1.37 (1.28, 1.46)
High	1.16 (1.06, 1.26)	1.17 (1.08, 1.28)	0.94 (0.85, 1.03)	0.96 (0.87, 1.05)	0.97 (0.87, 1.06)
Very high	1.00 (Reference)	1.00 (Reference)	1.00 (Reference)	1.00 (Reference)	1.00 (Reference)
Maternal age in years					
≤19		1.18 (1.04, 1.34)	1.15 (1.01, 1.32)	1.10 (0.96, 1.26)	1.10 (0.96, 1.26)
20–24		1.00 (Reference)	1.00 (Reference)	1.00 (Reference)	1.00 (Reference)
25–29		1.00 (0.89, 1.04)	1.02 (0.93, 1.10)	1.04 (0.96, 1.13)	1.04 (0.96 1.13)
30–34		0.94 (0.88, 1.01)	1.14 (1.06, 1.23)	1.22 (1.12, 1.32)	1.21 (1.12, 1.31)
35–39		1.14 (1.06, 1.22)	1.43 (1.32, 1.55)	1.60 (1.47, 1.73)	1.58 (1.45, 1.71)
≥40		1.48 (1.35, 1.62)	1.81 (1.64, 2.00)	2.03 (1.83, 2.24)	1.99 (1.79, 2.20)
Maternal education attainment					
Secondary education or lower			2.48 (2.31, 2.66)	2.56 (2.38, 2.75)	2.57 (2.39, 2.77)
Upper secondary or first stage of tertiary			1.21 (1.11, 1.31)	1.21 (1.11, 1.31)	1.21 (1.11, 1.31)
Tertiary education			1.00 (Reference)	1.00 (Reference)	1.00 (Reference)
Parity					
Nulliparous (first delivery)				1.31 (1.26, 1.37)	1.31 (1.26, 1.37)
Multiparous (≥1 deliveries)				1.00 (Reference)	1.00 (Reference)
Period 2007–15					
Per 1-year increase					1.02 (1.01, 1.03)

Notes: Model 1 univariate logistic model (outcome: stillbirth; exposure: 2015 HDI for maternal country of origin); Model 2 same as model 1 adjusted for age; Model 3 same as model 1 adjusted for age, maternal and education; Model 4 same as model 1 adjusted for age, maternal education and parity; Model 5 same as model 1 adjusted for age, maternal education, parity and period.

Overall, the joint effect of women with low educational attainment and low HDI increased approximately four times the risk of delivering a stillborn compared with women with tertiary education and very-high HDI. Similarly, the joint effect of advanced maternal age with low educational attainment increased four times the risk of delivering a stillborn compared with highly educated women aged between 25 and 29 years (Supplementary table S2).

## Discussion

We have found a consistent pattern of socioeconomic inequalities in the risk of delivering a stillborn in Spain during 2007–15, mainly characterized by a persistently higher stillbirth risk over time among women with lower SES (women from countries with low HDI, particularly Africa, and low educational attainment). Furthermore, we have found a paradoxical reduction of the stillbirth gap over time between the highest and the lowest SES mostly due to an increasing trend on stillbirth risk among highly educated women with advanced maternal age.

The persistent increased risk among women with lower SES is an important public health cause of concern in addition to the increasing stillbirth rates among highly educated women with advanced maternal age during the last 9-year period in Spain. Similar studies provide evidence of greater risk for stillbirth among women with low educational attainment.^3^ Furthermore, women living in regions with higher unemployment rates in Spain were shown to have twice the risk of stillbirth compared with women living in regions with less unemployment.^18^ However, the increasing trends in stillbirth among older women of higher education may reflect infertility problems associated with delayed childbearing and the use of assisted reproductive technologies.

Multiple studies have shown that lower SES can impact negatively pregnancy outcomes, whether due to differences in access to prenatal care or with regard to preferences about how to handle high-risk pregnancies.^19^ A systematic review examining pregnancy outcomes from immigrant women in western countries showed that women from Sub-Saharan African countries had higher perinatal mortality.^20^ Recently, a study showed similar findings in Brussels, Belgium.^21^ It has been described that social barriers from non-native foreign women can limit both access to antenatal screening and the ability to detect foetal growth restriction.^22^

To the best of our knowledge, this is the first study in Spain to demonstrate greater stillbirth risk among mothers of low SES. Furthermore, we improve the characterization of maternal SES adding to the maternal country of origin at birth two additional determinants of maternal SES: (i) HDI from the maternal country of origin at birth (women from countries, with low HDI,^14^ are more likely to have been exposed to adverse childhood environment^23^) (ii) and maternal educational attainment. It represents an improvement compared with other studies that only use maternal country of origin at birth allowing to better identify higher stillbirth risk groups.

We consider the migration phenomenon in Spain as a natural experiment given that immigration in Spain is an extremely recent phenomenon (late in the twentieth century), which means that an important number of foreign non-EU15 women included in the study are the first generations of economic migrants.^24^ Thus, the maternal country of origin at birth in Spain reflects, more precisely, on the maternal SES given the lower probability of acculturation. For instance, we argue that women from countries of low HDI and low educational attainment (i.e. women from some sub-Saharan countries) are less likely to have been misclassified as low SES.

Previous evidence in Spain showed that stillbirth rates declined from 1996 to 2006, whereas the risk increased among women of advanced maternal age.^25^ During the current period of analysis (2007–15) stillbirth rates remain constant (no secular trend), but the risk among highly educated women of advanced maternal age continued to increase. Furthermore, stillbirth rates among women from countries of low HDI remain constantly high showing no improvement during the last 10 years in Spain. We argue that public health policies banning preventive care for undocumented pregnant immigrant women during the 2009 economic crisis might explain the consistently higher rates of stillbirth among women of low SES in Spain. It is not only in Spain where undocumented immigrants have difficulties accessing healthcare. For instance, in Denmark, undocumented migrants have limited medical rights in addition to other barriers accessing healthcare such as arbitrariness in healthcare professionals' attitudes, fear of being reported to the police, poor language skills, lack of social network and lack of knowledge about the healthcare system.^26^ In general, undocumented migrants seem to use different types of healthcare services less often than legal residents in most of the European countries. Even when care is utilized, it often seems to be inadequate or insufficient.^27^

Our article extends the important research, which seeks to measure the association between the HDI from women’s countries of origin as a proxy for SES and identifying the joint effect between maternal educational attainment and HDI on stillbirth risk. However, to improve our understanding of the underlying causes of higher vulnerability to stillbirth among women with low educational attainment from countries with low HDI in Spain, more information related to immigrant background and culture, such as communication problems due to language skills, accessibility to the health-care system, acceptance of preventive interventions, use of prenatal services and quality of health care received, is needed.^28^ Understanding the mechanisms associated with these risk factors could have an important impact on the reduction of the stillbirth rates.

Stillbirth is particularly subjected to under-reporting at low gestational ages (20–27 weeks; early foetal death).^29^ However, the exclusion of infants <28 gestational weeks allowed us to minimize the bias due to under-reporting and misclassification of foetal deaths.^30^

Finally, we did not restrict our analysis to just singleton births, and we were not able to identify women delivering more than once during the period in study. It might have induced some confounding. However, sensitivity analysis restricted to singleton births (removing 168 900 multiple births) showed the same results as the unrestricted analysis.

Measuring social inequalities in stillbirth and monitoring trends over time enables target-setting for health policies. Unfortunately, our findings show no improvement in stillbirth rates among women with lower SES and an increasing trend among highly educated women with advanced maternal age in recent years in Spain. Therefore, the findings highlight the need for developing preventive programmes to reduce stillbirth rates among women with lower SES as well as the necessity of further study to understand the growing trend of age-related stillbirths among highly educated women in Spain.

## Funding

M.A.L.F. is supported by a Miguel Servet I Investigator Award (grant CP17/00206) from the Carlos III Institute of Health (ISCIII), Madrid, Spain. The findings and conclusions in this report are those of the authors and do not necessarily represent the views of the funding agencies. The corresponding author had full access to all the data in the study and had final responsibility for the decision to submit for publication.

*Conflicts of interest*: None declared.

**Key points**

- Despite the fact that in Europe stillbirth rates are lower than in developing countries, stillbirth rates remain relatively stable over the last few decades, which is the cause of public health concern.
- Furthermore, evidence shows a higher risk of stillbirth among women of lower socioeconomic status (SES) in Europe.
- The study shows no improvement in stillbirth rates, over the last decade, among women of lower SES in Spain.
- The findings highlight the need for developing preventive programmes to reduce stillbirth rates among women of lower SES.

## Supplementary Material

ckz086_Supplementary_Materials
