# Supplementary material for: Secular trends in stillbirth by maternal socioeconomic status in Spain 2007–15: a population-based study of 4 million births
Source: Eur J Public Health. 2019 May 23;29(6):1043–8. doi: 10.1093/eurpub/ckz086 (PMC6896972; doi:10.1093/eurpub/ckz086)
Supplement: ckz086_Supplementary_Materials [file ckz086_supplementary_materials.zip › ckz086-suppl_data/ejph-2018-07-om-0633-File006.docx]

**Supplementary Table 1.** Maternal age, education and country of origin by calendar period, 2007-2015 (11,323 stillbirths and 4,179,402 total births)

|  |  | 2007 | 2008 | 2009 | 2010 | 2011 | 2012 | 2013 | 2014 | 2015 |  |
| --- | --- | --- | --- | --- | --- | --- | --- | --- | --- | --- | --- |
| **Age** |  | n(%) | n(%) | n(%) | n(%) | n(%) | n(%) | n(%) | n(%) | n(%) | Total |
| ≤19 |  | 14,602(3.0) | 15,060(2.9) | 13,214(2.7) | 11,693(2.4) | 10,375(2.2) | 9,695(2.1) | 8,918(2.1) | 8,574(2.0) | 8,272(2.0) | 110,403(2.4) |
| 20-24 |  | 48,269(9.8) | 51,604(10.0) | 46,236(9.4) | 43,400(9.0) | 38,432(8.2) | 35,699(7.9) | 32,030(7.6) | 30,780(7.2) | 29,624(7.1) | 356,074(8.5) |
| 25-29 |  | 115,170(23.4) | 117,694(22.7) | 106,699(21.6) | 100,050(20.6) | 92,182(19.6) | 85,568(18.9) | 78,440(18.5) | 77,747(18.3) | 74,557(17.8) | 848,107(20.3) |
| 30-34 |  | 189,178(38.4) | 197,494(38.1) | 188,343(38.2) | 185,108(38.2) | 179,064(38.1) | 169,322(37.4) | 155,245(36.6) | 153,513(36.1) | 147,962(35.4) | 1,565,229(37.5) |
| 35-39 |  | 105,693(21.5) | 114,175(22.0) | 115,745(23.5) | 120,038(24.8) | 124,120(26.4) | 124,42(27.5) | 120,344(28.4) | 124,175(29.2) | 125,095(29.9) | 1,073,808(25.7) |
| $\geq$40 |  | 19,685(4.0) | 21,984(4.2) | 22,963(4.7) | 24,470(5.0) | 26,051(5.5) | 28,230(6.2) | 28,953(6.8) | 30,820(7.2) | 32,625(7.8) | 235,781(5.6) |
| **Education** |  |  |  |  |  |  |  |  |  |  |  |
| Secondary education or lower |  | 302,468(63.2) | 311,542(61.7) | 287,149(59.7) | 276,832(58.3) | 263,570(56.9) | 253,409(57.0) | 234,521(56.3) | 235,081(56.2) | 239,351(58.5) | 2,403,923(58.8) |
| Upper secondary or first stage of tertiary |  | 102,842(21.5) | 113,392(22.4) | 112,074(23.3) | 113,003(23.8) | 113,007(24.4) | 109,267(24.6) | 103,330(24.8) | 103,786(24.8) | 96,442(23.6) | 967,143(23.6) |
| Tertiary education |  | 73,224(15.3) | 80,276(15.9) | 81,742(17.0) | 84,792(17.9) | 86,735(18.7) | 81,928(18.4) | 78,558(18.9) | 79,448(19.0) | 73,237(17.9) | 719,940(17.6) |
| **Country of origin** |  |  |  |  |  |  |  |  |  |  |  |
| Spain |  | 399,211(81.0) | 411,760(79.5) | 392,546(79.6) | 386,655(79.8) | 380,022(80.8) | 367,463(81.1) | 346,780(81.8) | 351,421(82.6) | 345,044(82.5) | 3,380,902(80.9) |
| EU15 |  | 7,196(1.5) | 7,806(1.5) | 7,593(1.5) | 7,173(1.5) | 6,654(1.4) | 6,422(1.4) | 6,096(1.4) | 5,939(1.4) | 6,253(1.5) | 61,132(3.9) |
| Other European countries |  | 18,205(3.7) | 20,878(4.0) | 18,564(3.8) | 18,956(3.9) | 17,781(3.8) | 17,321(3.8) | 16,081(3.8) | 16,663(3.9) | 16,882(4.0) | 161,331(3.9) |
| Africa |  | 25,924(5.3) | 31,944(6.2) | 32,984(6.7) | 33,921(7.0) | 30,846(6.6) | 29,077(6.4) | 27,133(6.4) | 26,752(6.3) | 27,065(6.5) | 265,646(6.4) |
| America |  | 36,194(7.3) | 38,977(7.5) | 34,484(7.0) | 30,705(6.3) | 27,044(5.7) | 25,113(5.5) | 21,306(5.0) | 18,069(4.2) | 16,884(4.0) | 248,776(6.0) |
| Asia and Oceania |  | 5,867(1.2) | 6,646(1.3) | 7,029(1.4) | 7,349(1.5) | 7,877(1.7) | 7,541(1.7) | 6,534(1.5) | 6,765(1.6) | 6,007(1.4) | 61,615(1.5) |
|  |  |  |  |  |  |  |  |  |  |  |  |
