# Supplementary material for: Secular trends in stillbirth by maternal socioeconomic status in Spain 2007–15: a population-based study of 4 million births
Source: Eur J Public Health. 2019 May 23;29(6):1043–8. doi: 10.1093/eurpub/ckz086 (PMC6896972; doi:10.1093/eurpub/ckz086)
Supplement: ckz086_Supplementary_Materials [file ckz086_supplementary_materials.zip › ckz086-suppl_data/ejph-2018-07-om-0633-File007.docx]

| **Supplementary Table 2.** Joint effect of maternal age and 2015 human development index with maternal educational attainment on stillbirth risk in Spain, during 2007-2015 (11,323 stillbirths and 4,179,402 total births) |
| --- |

|  |  |  | **Human Development Index (2015)** | | | | | | | | | |  |
| --- | --- | --- | --- | --- | --- | --- | --- | --- | --- | --- | --- | --- | --- |
|  |  |  | Very-high |  | High | |  | Medium | |  | Low | |  |
|  |  |  | HDI: 1 - 0.8 |  | HDI: <0.8 - 0.7 | |  | HDI: <0.7 - 0.5 | |  | HDI: <0.55 - 0.8 | |  |
| **Maternal Education Attainment** | |  | RR (95% CI) |  | RR (95% CI) | |  | RR (95% CI) | |  | RR (95% CI) | |  |
| Secondary education or lower (I) | |  | 2.57 (2.39, 2.77) |  | 2.48 (2.21, 2.78) | |  | 3.52 (3.21, 3.86) | |  | 4.44 (3.71, 5.32) | |  |
| Upper secondary or first stage of tertiary (II) | |  | 1.21 (1.11, 1.31) |  | 1.17 (1.03, 1.33) | |  | 1.66 (1.49, 1.84) | |  | 2.10 (1.74, 2.53) | |  |
| Tertiary education (III) | |  | 1.0 (Reference) |  | 0.97 (0.87, 1.06) | |  | 1.37 (1.28, 1.46) | |  | 1.73 (1.46, 2.04) | |  |
|  |  |  | **Maternal age at delivery in years** | | | | | | | | | |  |
|  |  |  | 25-29 yr. |  | <25 yr. |  | | 30-34 yr. |  | | | ≥35 yr. | |
| **Maternal Educational Attainment** | |  | RR (95% CI) |  | RR (95% CI) |  | | RR (95% CI) |  | | | RR (95% CI) | |
| Secondary education or lower (I) | |  | 2.57 (2.39, 2.77) |  | 2.52 (2.27, 2.79) |  | | 3.00 (2.71, 3.30) |  | | | 4.08 (3.68, 4.51) | |
| Upper secondary or first stage of tertiary (II) | |  | 1.21 (1.11, 1.31) |  | 1.18 (1.06, 1.32) |  | | 1.41 (1.27, 1.56) |  | | | 1.91 (1.72, 2.12) | |
|  | Tertiary education (III) |  | 1.0 (Reference) |  | 0.98 (0.91, 1.05) |  | | 1.16 (1.10, 1.23) |  | | | 1.58 (1.49, 1.68) | |
|  |  |  |  |  |  |  | |  |  | | |  | |
