# Supplementary figures and images for: Secular trends in stillbirth by maternal socioeconomic status in Spain 2007–15: a population-based study of 4 million births
Source: Eur J Public Health. 2019 May 23;29(6):1043–8. doi: 10.1093/eurpub/ckz086 (PMC6896972; doi:10.1093/eurpub/ckz086)

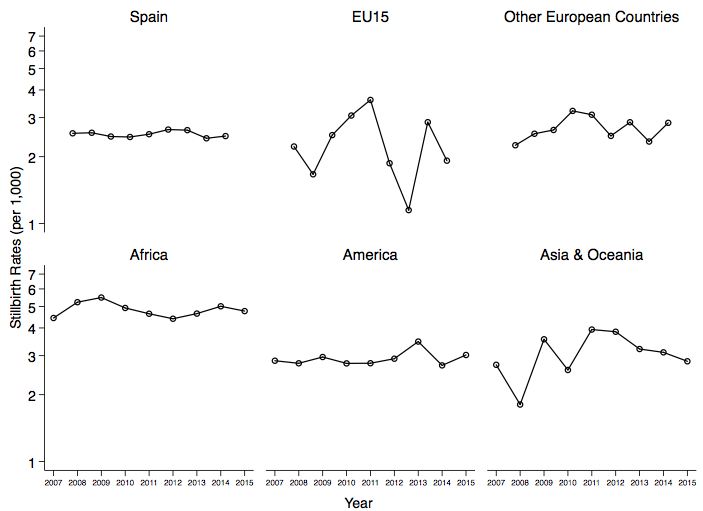

Supplement: ckz086_Supplementary_Materials [file ckz086_supplementary_materials.zip › ckz086-suppl_data/ejph-2018-07-om-0633-File005.tif]
